# Assessment of Anxiety and Depression Symptoms Among Medical Students and Their Association with Religiosity: A Cross-Sectional Study

**DOI:** 10.3390/diagnostics16010172

**Published:** 2026-01-05

**Authors:** Stipe Vidović, Zenon Pogorelić, Marija Olujić, Ana Pešikan, Ena Kolak, Ivana Groznica, Marija Jelić Vuković, Dubravka Biuk, Marija Heffer, Dunja Degmečić

**Affiliations:** 1 Faculty of Medicine Osijek, Josip Juraj Strossmayer University of Osijek, 31000 Osijek, Croatia; 2 Clinic for Eye Diseases, Clinical Hospital Centre Osijek, 31000 Osijek, Croatia; 3 Department of Surgery, School of Medicine, University of Split, 21000 Split, Croatia; 4 Department of Pediatric Surgery, University Hospital of Split, 21000 Split, Croatia; 5 Department for Medical Biology and Genetics, Faculty of Medicine Osijek, Josip Juraj Strossmayer University of Osijek, 31000 Osijek, Croatia; 6 Department of Psychiatry and Psychological Medicine, Faculty of Medicine Osijek, Josip Juraj Strossmayer University of Osijek, 31000 Osijek, Croatia; 7 Psychiatric Clinic, Clinical Hospital Centre Osijek, 31000 Osijek, Croatia

**Keywords:** anxiety, depression, religious and spiritual beliefs, mental health, mental disorders, suicidal ideation, student, medical student

## Abstract

**Background:** Depression and anxiety are highly prevalent among medical students and may adversely affect academic functioning and professional development. Early identification of these symptoms and their associated factors is therefore essential. Religiosity has been suggested as a potential modifier of mental health outcomes, although existing evidence remains inconsistent. This study aimed to assess symptoms of depression and anxiety, levels of religiosity, and their associations among medical students. **Methods:** This cross-sectional study was conducted among medical students at the University of Osijek, Croatia, from 29 September to 5 October 2025. Anxiety symptoms were assessed using the Generalized Anxiety Disorder-7 (GAD-7) scale, depressive symptoms using the Patient Health Questionnaire-9 (PHQ-9), and religiosity using the Duke University Religion Index (DUREL). **Results:** A total of 260 students (197 females, 63 males) completed the survey. At least mild depressive and anxiety symptoms were reported by 54.2% and 58.1%, respectively; 9.2% had moderately severe or severe depression, and 25% reported moderate to severe anxiety. Suicidal or self-harm ideation in the past two weeks occurred in 12.8%. Female students had higher median depression (*p* = 0.01) and anxiety scores (*p* = 0.005) than male students; however, these differences did not remain statistically significant after Bonferroni correction. Religiosity was not associated with PHQ-9 or GAD-7 scores after correction; however, categorical analyses indicated that students with moderate to high religiosity more frequently reported moderate to severe depressive symptoms (*p* = 0.037). **Conclusions:** The high prevalence of depression, anxiety, and suicidal ideation underscores the need for continuous mental health screening, early identification of risk factors, and implementation of preventive programs among the student population, particularly medical students as future healthcare professionals.

## 1. Introduction

Mental health disorders represent a major global public health concern, with depression and anxiety ranking among the most prevalent and disabling conditions worldwide [1]. According to the World Health Organization (WHO), over 300 million people worldwide are affected by depression, and approximately 4% of the global population experiences an anxiety disorder [2]. These conditions significantly reduce quality of life, impair daily functioning, and contribute to substantial social and economic burden across societies [3]. Early identification and systematic mental health screening have therefore emerged as crucial public health priorities, allowing timely detection of individuals at risk and facilitating preventive or therapeutic interventions before full clinical manifestation occurs [2].

Entering university marks a period of profound lifestyle and psychological transition for many young adults. Students must adapt to new academic environments, social networks, and living arrangements while simultaneously developing independence and managing multiple personal and professional responsibilities [4]. During this transition, maintaining healthy routines often becomes challenging, as fluctuations in workload and social commitments may disrupt sleep, nutrition, and physical activity [5,6]. Among university populations, medical students appear particularly vulnerable to mental health difficulties. The demanding nature of medical education—characterized by high academic expectations, long study hours, and emotionally taxing clinical experiences—places students under persistent psychological strain [7,8]. Balancing theoretical coursework with clinical obligations frequently leads to fatigue, irregular schedules, and limited opportunities for rest or recreation. Cumulatively, these stressors contribute to an elevated risk of anxiety and depressive symptoms commonly observed in this group [9,10,11]. These patterns underscore the importance of continuous monitoring of mental-health symptoms among medical students.

Recent evidence confirms these concerns. A meta-analysis by Daniali et al. (2023) reported that university students experience significantly higher levels of depressive and anxiety symptoms than the general population [12]. Similarly, an umbrella review by Paiva et al. (2025), encompassing 62 meta-analyses and 1655 studies, found that more than one-third of students worldwide exhibit depressive symptoms and over two-fifths report anxiety symptoms [13]. However, findings regarding differences between medical and non-medical students remain inconsistent [14,15,16,17,18,19], reflecting the influence of institutional, cultural, and methodological factors across studies. These inconsistencies highlight the need for further investigation using reliable screening tools in student populations.

In Croatia, a notable prevalence of depressive and anxiety symptoms among medical students was reported during the COVID-19 pandemic [20]. Subsequent cross-sectional studies conducted after the pandemic among medical students at the Faculty of Medicine Osijek likewise confirmed elevated symptom levels in this population [21,22,23]. These studies identified a bidirectional association with sleep quality and physical activity: poorer sleep quality, as measured by the Pittsburgh Sleep Quality Index (PSQI), was associated with higher levels of depression and anxiety, whereas lower physical activity and increased sedentary time, as measured by the International Physical Activity Questionnaire—Short Form (IPAQ-SF), were linked to greater symptom severity [22,23]. Sex-specific differences were also observed, with female students reporting higher levels of depression and anxiety than their male counterparts [22,23]. Despite these insights, neither previous studies conducted among medical students at the University of Osijek nor Croatian studies more broadly have simultaneously examined psychological symptoms alongside psychosocial or cultural correlates—such as religiosity—using standardized and validated mental-health screening tools.

Religiosity refers to adherence to organized beliefs, values, and practices related to a higher power [24]. Previous research suggests that religiosity may influence mental health through multiple pathways, including coping strategies, emotional regulation, meaning-making processes, and access to social support [25,26]. Although many studies report an inverse association between religiosity and symptoms of depression and anxiety [27,28,29], this relationship is not consistent across populations and contexts. For some individuals, religiosity may serve as a source of comfort, structure, and resilience during periods of stress, whereas for others it may evoke internal guilt, fear of divine punishment, or moral conflict—factors that have been linked to increased psychological distress [26,30,31]. Contemporary models distinguish between positive religious coping (e.g., seeking spiritual support, reframing stressful experiences as meaningful) and negative religious coping (e.g., spiritual struggle, perceived abandonment or punishment), with only the former consistently associated with better mental health outcomes [26,32]. Beyond individual coping styles, religiosity may also exert its effects through social mechanisms. Participation in religious communities can provide structured social support, a sense of belonging, and shared values, which may protect against loneliness and psychological distress [32]. In addition, religiosity has been associated with adaptive coping styles, resilience, and healthier behavioral routines, potentially reducing vulnerability to anxiety and depressive symptoms [33]. However, religiosity does not uniformly function as a protective factor. Certain forms of religious engagement—particularly those characterized by rigid moral norms, internalized guilt, fear of punishment, or unresolved spiritual conflict—have been associated with poorer mental health outcomes [34]. The impact of religiosity on mental health therefore appears to be context-dependent and shaped by individual differences as well as sociocultural norms. In predominantly Catholic societies such as Croatia, religiosity may be intertwined with social expectations and moral standards, potentially intensifying internal conflict when individuals perceive themselves as failing to meet religious or personal ideals [35]. Given the high psychological burden associated with medical training, it remains unclear whether religiosity among medical students functions primarily as a protective resource or, conversely, as an additional source of emotional strain. Examining this relationship may thus provide important insights into coping processes during medical education.

To the best of our knowledge, this is the first study in Croatia to comprehensively assess depressive and anxiety symptoms, religiosity, and a broad range of sociodemographic and lifestyle characteristics among medical students at the Faculty of Medicine Osijek using standardized and validated screening instruments. In addition, we examined sex-specific differences and explored associations between mental health outcomes and levels of religiosity.

## 2. Materials and Methods

### 2.1. Study Design and Participants

This was a cross-sectional study conducted among medical students at the Faculty of Medicine Osijek, University of Osijek, Croatia. All medical students from this faculty were eligible to participate, and no exclusion criteria were applied other than non-completion of the questionnaire.

### 2.2. Study Settings and Method of Recruitment

Recruitment for this cross-sectional study was conducted exclusively via official institutional email addresses. On 29 September 2025, an invitation email was distributed to all eligible students enrolled in the medical program at the Faculty of Medicine Osijek, University of Osijek, Croatia. The email included an information sheet outlining the study objectives, methodology, eligibility criteria, and estimated participation time, as well as details on potential benefits and risks. Participation was entirely voluntary and anonymous, with assurances that declining or withdrawing would have no academic or personal consequences. No identifying data (e.g., name, student ID, or email address) were collected, and all responses were stored securely and used solely for research purposes. Participants provided electronic informed consent before accessing the questionnaire, which was available from 29 September to 5 October 2025, via a secure Google Forms survey link. To minimize missing data, the Google Forms platform was configured such that all items were mandatory prior to submission; therefore, the final dataset contained no missing values. Supplement File S1 contains the Participant Information Sheet, and Supplement File S2 provides the Informed Consent Form. The described recruitment methodology is consistent with procedures employed in our previous research [22,23].

### 2.3. Questionnaire

The questionnaire consisted of 40 items in Croatian and required approximately 10 min to complete. It was divided into four sections. The English translation of the questionnaire is provided in Supplement File S2.

The first section assessed sociodemographic characteristics, including age, sex, year of study, grade point average (GPA), place of residence, living arrangements, relationship status, financial/material situation, parental education, and consumption of alcohol, cigarettes, and psychoactive substances. The sociodemographic questions were adapted from instruments used in our previous studies among medical students to ensure comparability of data [22,23].

The second section evaluated anxiety symptoms using the self-reported Generalized Anxiety Disorder-7 (GAD-7) instrument. GAD-7 is a brief and reliable instrument commonly used to assess and monitor the severity of anxiety symptoms. It comprises seven items that measure the frequency of anxiety-related experiences over the past two weeks, rated on a four-point Likert scale ranging from 0 (“not at all”) to 3 (“nearly every day”). The total score ranges from 0 to 21, with higher scores indicating greater levels of anxiety. Based on the total score, respondents were classified into four categories of anxiety severity: minimal (0–4), mild (5–9), moderate (10–14), and severe (15–21). In the present study, the Croatian version of the GAD-7 was used [36,37,38]. The GAD-7 showed a Cronbach’s alpha coefficient of 0.90.

The third section assessed depressive symptoms using the self-reported Patient Health Questionnaire-9 (PHQ-9). PHQ-9 is a widely used, validated instrument designed to assess the severity of depressive symptoms. It consists of nine items that correspond to the diagnostic criteria for major depressive disorder, based on the DSM-IV. Respondents rate how often they have been bothered by each symptom over the past two weeks, using a four-point Likert scale ranging from 0 (“not at all”) to 3 (“nearly every day”). The total score ranges from 0 to 27, with higher scores indicating more severe depressive symptoms. Based on the total score, participants are categorized into five levels of depression severity: minimal (0–4), mild (5–9), moderate (10–14), moderately severe (15–19), and severe (20–27). In this study, the Croatian version of the PHQ-9 was used [38,39,40]. The PHQ-9 showed a Cronbach’s alpha coefficient of 0.88.

The fourth section assessed religious involvement by quantifying different aspects of religiosity using the Duke University Religion Index (DUREL). The DUREL consists of five items assessing three dimensions: organized religious activity (ORA), non-organized religious activity (NORA), and intrinsic religiosity (IR). The first two items are scored on a six-point scale (1 = “never” to 6 = “more than once a week”), and the remaining three on a five-point scale (1 = “definitely not true” to 5 = “definitely true”). Higher scores indicate greater religiosity. Total scores (range 5–27) were categorized as low (5–12), moderate (13–19), or high (20–27) religiosity. In this study, the validated Croatian version of the DUREL was used [41,42]. The internal consistency of the DUREL was assessed using Cronbach’s alpha, which yielded α = 0.90.

### 2.4. Sample Size

The minimum required sample size (*n*) was calculated for a population proportion using a 95% confidence level (*Z* = 1.96), a margin of error of ±5% (*E* = 0.05), and an assumed population proportion of *p* = 0.5. Because *p* = 0.5 maximizes the variance term *p*(1 − *p*), it results in the largest required sample size and therefore represents the most conservative assumption when the true population proportion is unknown. Applying the finite population correction for the total population (*N* = 444), the adjusted minimum required sample size was approximately 206 participants, as determined by the following formula:

$$n=\frac{N\times Z^{2}\times p\times (1-p)}{E^{2}\times (N-1)+Z^{2}\times p\times (1-p)}$$

### 2.5. Statistical Analysis

Data distribution was assessed for normality using the Shapiro–Wilk test. As most continuous variables deviated from a normal distribution, non-parametric statistical methods were applied throughout the analyses. Descriptive statistics were used to summarize sociodemographic and lifestyle characteristics, depressive symptoms assessed by the Patient Health Questionnaire-9 (PHQ-9), anxiety symptoms assessed by the Generalized Anxiety Disorder-7 (GAD-7), and religiosity dimensions measured using the Duke University Religion Index (DUREL). Categorical variables are presented as frequencies and percentages, while continuous variables are reported as medians with interquartile ranges (IQRs). Differences in PHQ-9 and GAD-7 scores between two independent groups were examined using the Mann–Whitney U test. Comparisons across three or more independent groups were performed using the Kruskal–Wallis H test. For Kruskal–Wallis tests yielding statistically significant results, post hoc pairwise comparisons were conducted using the Mann–Whitney U test with Bonferroni-adjusted significance thresholds, calculated by dividing the nominal alpha level (0.05) by the number of pairwise comparisons. To control for multiple testing across sociodemographic and lifestyle variables in the primary analyses, a Bonferroni-adjusted significance threshold was applied (adjusted α = 0.003). Effect sizes are reported as *r* for Mann–Whitney U tests and eta squared (η^2^) for Kruskal–Wallis tests. Associations between categorical variables, including depressive and anxiety symptom severity categories and religiosity level, were analyzed using the chi-squared (χ^2^) test. Relationships between continuous PHQ-9 scores, GAD-7 scores, and DUREL component scores were assessed using Spearman’s rank correlation coefficients (ρ). Statistical significance was set at *p* < 0.05 unless otherwise adjusted for multiple comparisons. Statistical analyses were conducted using IBM SPSS Statistics for Windows (version 26.0; IBM Corp., Armonk, NY, USA).

### 2.6. Raw Data

The raw data collected in the study, without any indirect identifiers of the participants, are available in Supplement File S3.

## 3. Results

Of the 444 medical students invited to participate (300 female, 144 male), 260 completed the survey (197 female, 63 male), yielding an overall response rate of 59.2% (61.0% among female and 53.5% among male students). The achieved sample size met the minimum statistical requirements.

The study sample consisted of more female students (*n* = 197) than male students (*n* = 63). The majority of participants (59.2%) were between 18 and 21 years of age. Additional sociodemographic characteristics of the participants are presented in Table 1.

According to the PHQ-9 questionnaire, 54.2% of respondents had at least mild symptoms of depression, while 9.2% had moderately severe or severe symptoms of depression. Furthermore, according to the GAD-7 questionnaire, 58.1% of respondents had at least mild symptoms of anxiety, whereas 25% had moderate or severe symptoms of anxiety. The distribution of depressive and anxiety symptoms across different categories is presented in Table 2.

The ninth item of the PHQ-9 questionnaire assesses how often, over the past two weeks, respondents experienced thoughts that they would be better off dead or of hurting themselves in some way. A total of 12.8% of respondents reported having such thoughts on at least several days during the past two weeks. The distribution of responses is presented in Table 3.

In response to the question on whether professional advice and support from a psychologist or psychiatrist would be beneficial considering their current mental health, 100 respondents (38.5%) answered affirmatively. Regarding the question of whether they had sought help from a psychologist or psychiatrist, 44 respondents (16.9%) reported that they had.

The distribution of responses to the DUREL components, including Organizational Religious Activity (ORA), Non-Organizational Religious Activity (NORA), and Intrinsic Religiosity (IR), is presented in Table 4. Based on the total DUREL score, 28.1% of respondents were classified as having low religiosity, 31.5% as moderate religiosity, and 40.4% as high religiosity.

Median GAD-7 scores were compared across sociodemographic, academic, and lifestyle characteristics. In unadjusted analyses, GAD-7 scores differed by sex, with female students reporting higher anxiety levels than male students (*p* = 0.005), by age group (*p* = 0.031), and by year of study (*p* = 0.024). However, none of these associations remained statistically significant after correction for multiple comparisons (adjusted α = 0.003). No other sociodemographic, academic, or lifestyle variables—including BMI, GPA, place of residence, living arrangement, material/financial status, parental education, smoking status, psychoactive substance use, or religiosity—were significantly associated with GAD-7 scores after correction (Table 5).

Median PHQ-9 scores were compared across sociodemographic, academic, and lifestyle characteristics. In unadjusted analyses, PHQ-9 scores differed by sex, with female students reporting higher median scores than male students (*p* = 0.010); however, this association did not remain statistically significant after Bonferroni correction for multiple comparisons (adjusted α = 0.003). No statistically significant differences in PHQ-9 scores were observed across other sociodemographic, academic, or lifestyle variables, including age, BMI, year of study, GPA, place of residence, living arrangement, material/financial status, parental education, smoking status, psychoactive substance use, or religiosity (Table 6).

In a focused sex-specific analysis (Table 7), female students exhibited higher median scores on both the GAD-7 (*p* = 0.005) and PHQ-9 (*p* = 0.01) compared with male students. No significant sex differences were observed for ORA, NORA, IR, or total DUREL scores.

When depressive and anxiety symptoms were analyzed categorically, religiosity level was significantly associated with depressive symptom severity (χ^2^ = 4.36, *p* = 0.037), with participants reporting moderate to high religiosity more frequently classified into moderate to severe depression categories compared with those with low religiosity. In contrast, religiosity was not significantly associated with anxiety symptom categories (χ^2^ = 0.77, *p* = 0.381) (Table 8).

Depression and anxiety scores were not significantly associated with any of the DUREL components (*p* > 0.05) (Table 9).

## 4. Discussion

Using validated screening instruments (PHQ-9, GAD-7, and DUREL), this study identified a high prevalence of anxiety and depressive symptoms among medical students at the University of Osijek, with a notable proportion of students reporting severe anxiety or moderately severe to severe depressive symptoms; female students exhibited higher levels of both. Notably, approximately one in ten respondents endorsed thoughts of death or self-harm. While religiosity was not independently associated with depression or anxiety scores after correction for multiple testing, students with moderate to high religiosity were more likely to report moderate to severe depressive symptom categories compared with those reporting low religiosity.

In this study, a substantial proportion of medical students reported symptoms of depression and anxiety. Comparable prevalence rates among medical and nursing students in Croatia were reported by Milić et al. (2019), who, using the PHQ-9 and GAD-7 instruments, found that 60.2% and 54.2% of respondents, respectively, exhibited at least mild symptoms of depression and anxiety [21]. During the COVID-19 pandemic, Milić et al. (2024) conducted a multicenter study across 10 higher education institutions in Croatia employing the same instruments, and observed a lower prevalence of depressive symptoms (41.4%) but a higher prevalence of anxiety symptoms (67.5%) [38]. Following the pandemic, Vidović et al. (2025), using the Depression Anxiety and Stress Scale 21 (DASS-21) among medical students, reported lower prevalence rates, with 38.8% of respondents experiencing at least mild depressive symptoms and 45.3% reporting at least mild anxiety symptoms [22]. Taken together, these studies consistently highlight the high prevalence of depression and anxiety symptoms among medical students. In line with these findings, our study revealed that nearly one in ten respondents self-reported moderately severe to severe depressive symptoms, while approximately one-fifth reported moderate to severe anxiety symptoms. It should also be noted that a substantial proportion of our sample consisted of first-year medical students, who may not yet be fully accustomed to the medical education system, which could partly influence the levels of depression and anxiety observed. The consistently high prevalence of depressive and anxiety symptoms among medical students may be attributed to the demanding nature of medical education, including academic overload, frequent examinations, and exposure to emotionally challenging clinical experiences [43,44]. Additional contributing factors may include insufficient availability of mental health resources, high levels of competitiveness, and reduced opportunities for rest and recovery [9,45]. The observed fluctuations in prevalence across different studies, particularly during and after the COVID-19 pandemic, suggest that external stressors and broader societal circumstances also play a significant role in shaping students’ mental health outcomes [46].

In our study, female students reported higher levels of depression and anxiety compared to their male counterparts. Previous research conducted in our country that examined gender-specific differences in these outcomes has likewise found that women tend to exhibit higher levels of depressive and anxiety symptoms [22,23,38]. These findings are in line with the meta-analysis by Daniali et al. (2023), which demonstrated that females consistently report greater symptom severity in both domains, as well as with the systematic review and meta-analysis by Ahmed et al. (2023), which concluded from 89 studies that being female is associated with higher levels of anxiety [12,47]. Several mechanisms may underlie the higher levels of depression and anxiety observed among female students. Women are generally more prone to internalizing distress and more likely to acknowledge and report emotional symptoms, while biological factors such as hormonal fluctuations, heightened hypothalamic–pituitary–adrenal (HPA) axis reactivity, and serotonergic differences contribute to increased vulnerability [12,48,49]. Moreover, female students often face compounded psychosocial pressures, balancing academic and social expectations, and may rely more on ruminative coping styles, which can sustain negative affect [50,51]. Finally, two cross-sectional studies conducted among medical students in Osijek indicated that female students reported poorer sleep quality, greater sedentary behavior, and lower levels of physical activity, all of which are established predictors of depression, anxiety, and stress and may further contribute to the observed gender differences [22].

A noteworthy finding of this study is that slightly more than one tenth of respondents reported having thoughts of death or self-harm during the previous two weeks. This observation is particularly concerning given recent evidence indicating an increase in suicidality among adolescents aged 15–19 years in Croatia [52]. Moreover, more than one third of participants indicated that, given their current mental health status, they would benefit from professional psychological or psychiatric support. These findings underscore the importance of continued research into mental health and associated risk factors, the insights from which could inform the development of targeted public health strategies and interventions aimed at improving mental well-being and reducing suicidal ideation among young adults. In this context, the routine use of brief, validated screening instruments such as the PHQ-9 and GAD-7 could facilitate early identification of students experiencing distress, enabling timely referral to appropriate mental health services and supporting evidence-based prevention strategies within university settings [36,37,39,53]. In addition to early identification, universities could play an important role in supporting student mental health by implementing campus-based counselling services, structured psychoeducation programs on stress management and healthy coping strategies, and resilience- or mindfulness-based interventions within the curriculum [54,55]. Furthermore, in the Croatian context, where Catholicism represents the predominant religious affiliation, optional programs that promote healthy forms of spirituality—such as compassion, community support, forgiveness, and a sense of meaning and belonging—consistent with values emphasized within Catholic teaching, may offer an additional source of comfort for students who draw on their faith during stressful periods [35,56].

The results of our study indicate a nuanced relationship between religiosity and depressive symptoms among medical students. Analyses based on symptom categories showed that students with moderate to high religiosity more frequently reported moderate to severe depressive symptoms, whereas comparisons based on continuous PHQ-9 scores did not remain statistically significant after correction for multiple testing. Similar findings have been reported in previous research, suggesting that religiosity does not uniformly function as a protective factor for mental health. Aggarwal et al. (2023), for example, emphasized that internal religious guilt or doubt, as well as feelings of being abandoned or punished by God, may contribute to the exacerbation of depressive symptoms [26]. At the same time, the literature also documents numerous cases in which religiosity serves as a buffer against psychological distress. Bonelli et al. (2012) and Forouhari et al. (2019) reported that, for many individuals, religious faith and practice may foster meaning-making, resilience, and healthier coping strategies, thereby reducing depressive and anxiety symptoms [57,58]. These contrasting findings underscore that the relationship between religiosity and mental health is not linear but complex and multifaceted, shaped by sociocultural context and individual differences in how religiosity is internalized and expressed. In the Croatian sociocultural environment, where Catholicism is the predominant religious tradition, religiosity may also carry elements of social expectation and moral conformity. Students who strongly identify with religious norms may experience psychological conflict when they perceive themselves as failing to meet moral or spiritual standards, leading to internalized guilt and self-criticism [59,60]. In such cases, religiosity may not provide emotional relief but instead become an additional source of stress, particularly for individuals with perfectionistic tendencies or rigid cognitive styles. These dynamics may help explain why higher religiosity coincided with greater depressive symptomatology among a subset of students in our study. Taken together, the existing evidence—including our findings—highlights the need to move beyond treating religiosity as uniformly protective or uniformly detrimental [61]. Instead, religiosity should be conceptualized as a multidimensional construct that can promote emotional resilience for some individuals while contributing to psychological burden for others, depending on cultural context, coping style, and personal meaning systems [26]. Future research should therefore aim to identify which forms and expressions of religiosity contribute to adaptive versus maladaptive coping in medical student populations.

From a theoretical standpoint, these findings support an emerging body of evidence suggesting that religiosity should not be conceptualized as a uniformly protective factor for mental health in young adults; rather, its influence may vary depending on sociocultural context and the nature of religious coping strategies [62]. From a practical perspective, the results highlight the need for universities to integrate systematic mental-health screening into student support systems and to strengthen access to campus-based psychological services [63]. In addition, voluntary programs that promote adaptive coping and healthy forms of spiritual engagement may be particularly beneficial for students who draw on their faith during periods of distress [64].

### Limitations and Further Directions

This study has several limitations that should be considered when interpreting the findings. First, although the sample size met minimum statistical requirements, it may not have been sufficient to detect small effect sizes, potentially leading to type II errors. Moreover, the study was conducted among medical students from a single university, which limits the generalizability of the results to other medical faculties or student populations in Croatia. The lack of available sociodemographic and academic data on non-responders further precluded assessment of potential response bias. Future research should consider a multicenter approach that includes medical students from other Croatian universities, such as those in Split, Zagreb, and Rijeka. Expanding the sample to include students from other academic disciplines would also provide a more comprehensive understanding of the phenomena under investigation.

Second, the cross-sectional design prevents causal inference and limits conclusions regarding the directionality of observed associations. In particular, it cannot be determined whether religiosity influenced depressive or anxiety symptoms or whether students experiencing psychological distress were more likely to engage in religious coping.

Third, depressive and anxiety symptoms were assessed exclusively using self-reported screening instruments (PHQ-9 and GAD-7). Although these tools are well validated and widely used, they do not establish clinical diagnoses and may be affected by recall bias, social desirability, or underreporting of sensitive symptoms. Future research could strengthen validity by incorporating structured clinical interviews alongside self-report measures.

Finally, while religiosity was assessed using the DUREL scale, other relevant psychosocial, academic, and lifestyle factors—such as academic workload, physical activity, sleep quality, metabolic disturbances related to dietary composition, perceived social support, social media use and internet addiction, childhood trauma, and personality traits—were not systematically evaluated. Longitudinal studies integrating these variables would provide a more comprehensive understanding of the complex determinants of mental health and suicidality among university students and support the development of targeted preventive interventions.

From a statistical perspective, several sociodemographic and lifestyle categories included a very small number of participants (e.g., older age groups, extreme BMI categories, and lower parental education levels), which may have reduced the statistical power of nonparametric comparisons and increased the risk of Type II error. Additionally, the application of Bonferroni correction, while reducing the likelihood of Type I error, may have further limited the ability to detect true differences. Therefore, the absence of statistically significant findings in these categories should be interpreted with caution.

## 5. Conclusions

This study highlights the need for systematic mental-health screening and targeted support programs for medical students. The observed association between religiosity and depressive symptoms underscores the importance of further research into the cultural and psychosocial dimensions of coping.

## Figures and Tables

**Table 1:** Sociodemographic characteristics of participants (*n* = 260).

Variables		*n*
Sex	Male	63 (24.2%)
	Female	197 (75.8%)
Age	18–21	154 (59.2%)
	22–25	100 (38.5%)
	26–29	4 (1.5%)
	>30	2 (0.8%)
BMI	<18.5	18 (6.9%)
	18.5–24.9	181 (69.6%)
	25.0–29.9	55 (21.2%)
	30.0–34.9	3 (1.15%)
	35.0–39.9	3 (1.15%)
Year of study	Preclinical studies (First, second and third year of study)	163 (62.7%)
	Clinical studies (Fourth, fifth, and sixth year of study)	97 (37.3%)
GPA *	2–3	1 (0.4%)
	3.1–4	61 (23.5%)
	4.1–5	198 (76.1%)
Place of residence	Urban	187 (71.9%)
	Rural	73 (28.1%)
Living	Alone	50 (19.2%)
	With roommate	100 (38.5%)
	With family	110 (42.3%)
Material/financial status	Far below average	8 (3.1%)
	Below average	15 (5.8%)
	Average	124 (47.7%)
	Above average	100 (38.5%)
	Far above average	13 (5%)
Relationship status	Single	140 (53.8%)
	In relationship	120 (46.2%)
Fathers’ education	Primary education	3 (1.2%)
	Secondary education	144 (55.4%)
	Higher/vocational	113 (43.5%)
Mothers’ education	Primary education	8 (3.1%)
	Secondary education	136 (52.3%)
	Higher/vocational	116 (44.6%)
Your parents live	Together	215 (82.7%)
	Not together	45 (17.3%)
Smoking	Current smoker	43 (16.5%)
	Ex-smoker	13 (5%)
	Non-smoker	204 (78.5%)
Psychoactive substances	Yes/sometimes	9 (3.5%)
	No	251 (96.5%)

BMI = Body mass Index; GPA = Grade Point Average. * GPA in Croatia is calculated as the arithmetic mean of all positive course grades. The grading scale ranges from 2 to 5 (2 = pass, 3 = good, 4 = very good, 5 = excellent), while 1 = fail is not included in GPA calculations. For reporting purposes, GPA was grouped into three categories: 2.0–3.0, 3.1–4.0, and 4.1–5.0.

**Table 2:** Prevalence of anxiety symptoms (GAD-7) and depression symptoms (PHQ-9) among medical students (*n* = 260).

Symptoms	Severity	*n*
Depression	Minimal	119 (45.8%)
	Mild	85 (32.7%)
	Moderate	32 (12.3%)
	Moderately severe	17 (6.5%)
	Severe	7 (2.7%)
Anxiety	Minimal	109 (41.9%)
	Mild	86 (33.1%)
	Moderate	41 (15.8%)
	Severe	24 (9.2%)

**Table 3:** Distribution of responses to PHQ-9 Item 9 assessing self-harm and suicidal thoughts (*n* = 260).

PHQ-9 Item 9	Answers	*n*
Over the last 2 weeks, how often have you been bothered by any of the following problems? Thoughts that you would be better off dead or of hurting yourself in some way	Not at all	227 (87.3%)
	Several days	22 (8.5%)
	More than half the days	8 (3.1%)
	Nearly every day	3 (1.2%)

**Table 4:** The Duke University Religion Index (DUREL) components, corresponding items, and distribution of responses among medical students (*n* = 260).

DUREL Components (Question/Statement)	Answer	*n*
Organizational religious activity (ORA)		
1. How often do you attend a church/temple/mosque, etc., or other religious meetings?	More than once a week	17 (6.5%)
	Once a week	80 (30.8%)
	Two or three times a month	33 (12.7%)
	A few times a year	52 (20%)
	Once a year or less	35 (13.5%)
	Never	43 (16.5%)
Non-organized religious activity (NORA)		
1. How often do you spend time in private religious activities, such as prayer, meditation, or Bible study?	More than once a day.	10 (3.8%)
	Daily	90 (34.6%)
	Two or three times a week	29 (11.2%)
	Once a week	21 (8.1%)
	A few times a month	22 (8.5%)
	Rarely or never	88 (33.9%)
Intrinsic religiosity (IR)		
1. In my life, I experience the presence of the Divine (i.e., God)	Definitely true of me	94 (36.2%)
	Tends to be true	61 (23.5%)
	Unsure	48 (18.5%)
	Tends not to be true	21 (8.1%)
	Definitely not true	36 (13.8%)
2. My religious beliefs are what really lie behind my whole approach to life	Definitely true of me	52 (20%)
	Tends to be true	70 (26.9%)
	Unsure	53 (20.4%)
	Tends not to be true	36 (13.8%)
	Definitely not true	49 (18.8%)
3. I try hard to carry my religion over into all other dealings in life.	Definitely true of me	55 (21.2%)
	Tends to be true	79 (30.4%)
	Unsure	51 (19.6%)
	Tends not to be true	34 (13.1%)
	Definitely not true	41 (15.8%)

DUREL = The Duke University Religion Index.

**Table 5:** Differences in GAD-7 scores across sociodemographic and lifestyle characteristics (*n* = 260).

Variable	Category (*n*)	GAD-7 Score	Statistical Test	*p*	Effect Size
Sex	Male (63)	4 (1–8)	MWU	0.005	*r* = 0.18
	Female (197)	6 (3–10)			
Age	18–21 (154)	6 (3–10)	KW	0.031	*η*^2^ = 0.02
	22–25 (100)	4 (2–8)			
	26–29 (4) ^a^	8 (6–9)			
	≥30 (2) ^a^	4 (3–5)			
BMI	<18.5 (18)	6 (3–13)	KW	0.735	η^2^ < 0.001
	18.5–24.9 (181)	5 (3–9)			
	25–29.9 (55)	6 (3–9)			
	30–34.9 (3) ^a^	8 (6–12)			
	35–39.9 (3) ^a^	7 (7–11)			
Year of study	Preclinical studies (163)	6 (3–10)	MWU	0.024	*r* = 0.148
	Clinical studies (97)	5 (2–8)			
GPA	2–3 (1) ^a^	1 (1–1)	KW	0.413	*η*^2^ = 0.006
	3.1–4 (61)	5 (2–10)			
	4.1–5 (198)	5 (3–9)			
Place of residence	Urban (187)	5 (3–10)	MWU	0.298	*r* = 0.006
	Rural (73)	6 (3–9)			
Living	Alone (50)	5 (3–9)	KW	0.267	*η*^2^ = 0.002
	With roommate (100)	6 (3–10)			
	With family (110)	5 (3–9)			
Material status	Far below average (8)	10 (5–15)	KW	0.371	*η*^2^ = 0.004
	Below average (15)	6 (3–10)			
	Average (124)	5 (3–9)			
	Above average (100)	5 (2–8)			
	Far above average (13)	3 (2–6)			
Relationship status	Single (140)	5 (2–9)	MWU	0.138	*r* = 0.092
	In relationship (120)	6 (3–10)			
Fathers’ education	Primary (3) ^a^	5 (4–11)	KW	0.669	*η*^2^ = 0.003
	Secondary (144)	5 (3–9)			
	Higher/vocational (113)	6 (3–10)			
Mothers’ education	Primary (8)	4 (3–5)	KW	0.704	*η*^2^ = 0.002
	Secondary (136)	6 (3–9)			
	Higher/vocational (116)	5 (3–10)			
Your parents live	Together (215)	5 (3–9)	MWU	0.444	*r* = 0.047
	Not together (45)	6 (4–9)			
Smoking	Current smoker (43)	4 (2–10)	KW	0.21	*η*^2^ = 0.008
	Ex-smoker (13)	7 (6–12)			
	Non-smoker (204)	5 (3–9)			
Psychoactive substances	Yes/sometimes (9)	11 (4–13)	MWU	0.196	*r* = 0.080
	No (251)	5 (3–9)			
DUREL category	Low religiosity ^†^ (73)	5 (3–8)	MWU	0.163	*r* = 0.086
	Moderate and high religiosity ^‡^ (187)	6 (3–10)			

GAD-7 score = Generalized Anxiety Disorder 7-item scale score; MWU = Mann–Whitney U test; KW = Kruskal–Wallis H test; η^2^ = Eta squared; BMI = Body mass index; GPA = Grade Point Average. ^†^ = Respondents with a total DUREL score of 12 or lower; ^‡^ = Respondents with a total DUREL score of 13 or higher. Note: GAD-7 scores are presented as median (IQR). Effect sizes are reported as eta squared (η^2^) for the Kruskal–Wallis test (KW) and r for the Mann–Whitney U (MWU) test. ^a^ = Categories with small subsample sizes (*n* < 5), which may limit the robustness of the estimates.

**Table 6:** Differences in PHQ-9 scores across sociodemographic and lifestyle characteristics (*n* = 260).

Variable	Category (*n*)	PHQ-9 Score	Statistical Test	*p*	Effect Size
Sex	Male (63)	4 (2–9)	MWU	0.01	*r* = 0.187
	Female (197)	5 (3–9)			
Age	18–21 (154)	6 (3–9)	KW	0.093	*η*^2^ = 0.016
	22–25 (100)	4 (2–8)			
	26–29 (4) ^a^	8 (7–9)			
	≥30 (2) ^a^	6 (3–5)			
BMI	<18.5 (18)	6 (3–15)	KW	0.262	*η*^2^ = 0.007
	18.5–24.9 (181)	5 (2–9)			
	25–29.9 (55)	5 (2–9)			
	30–34.9 (3) ^a^	10 (8–15)			
	35–39.9 (3) ^a^	7 (5–11)			
Year of study	Preclinical studies (163)	5 (2–9)	MWU	0.242	*r* = 0.031
	Clinical studies (97)	4 (2–7)			
GPA	2–3 (1) ^a^	5 (5–5)	KW	0.636	*η*^2^ = 0.005
	3.1–4 (61)	6 (3–10)			
	4.1–5 (198)	5 (2–9)			
Place of residence	Urban (187)	5 (3–9)	MWU	0.620	*r* = 0.057
	Rural (73)	5 (3–9)			
Living	Alone (50)	5 (3–7)	KW	0.182	*η*^2^ = 0.014
	With roommate (100)	6 (3–10)			
	With family (110)	4 (2–9)			
Material status	Far below average (8)	10 (6–16)	KW	0.140	*η*^2^ = 0.014
	Below average (15)	5 (2–10)			
	Average (124)	4 (3–8)			
	Above average (100)	5 (3–9)			
	Far above average (13)	2 (2–10)			
Relationship status	Single (140)	5 (2–9)	MWU	0.217	*r* = 0.076
	In relationship (120)	5 (3–9)			
Fathers’ education	Primary (3) ^a^	6 (4–13)	KW	0.061	*η*^2^ = 0.026
	Secondary (144)	5 (2–9)			
	Higher/vocational (113)	5 (3–9)			
Mothers’ education	Primary (8)	3 (1–6)	KW	0.258	*η*^2^ = 0.010
	Secondary (136)	6 (3–9)			
	Higher/vocational (116)	5 (3–10)			
Your parents live	Together (215)	5 (2–9)	MWU	0.486	*r* = 0.060
	Not together (45)	6 (3–9)			
Smoking	Current smoker (43)	5 (2–6)	KW	0.099	*η*^2^ = 0.012
	Ex-smoker (13)	9 (5–9)			
	Non-smoker (204)	5 (3–9)			
Psychoactive substances	Yes/sometimes (9)	9 (5–12)	MWU	0.111	*r* = 0.099
	No (251)	5 (3–9)			
DUREL category	Low religiosity ^†^ (73)	4 (2–6)	MWU	0.018	*r* = 0.144
	Moderate and high religiosity ^‡^ (187)	6 (3–10)			

PHQ-9 score = Patient Health Questionnaire 9-item scale score; MWU = Mann–Whitney U test; KW = Kruskal–Wallis H test; BMI = Body mass index; GPA = Grade Point Average. ^†^ = Respondents with a total DUREL score of 12 or lower; ^‡^ = Respondents with a total DUREL score of 13 or higher. Note: PHQ-9 scores are presented as median (IQR). Effect sizes are reported as eta squared (η^2^) for the Kruskal–Wallis test (KW) and r for the Mann–Whitney U (MWU) test. ^a^ = Categories with small subsample sizes (*n* < 5), which may limit the robustness of the estimates.

**Table 7:** Sex-specific differences in anxiety (GAD-7), depression (PHQ-9), and Duke University Religion Index (DUREL) component scores (*n* = 260).

	Male (*n* = 63)	Female (*n* = 197)		
Variables	Median (IQR)	Median (IQR)	U	*p*
Anxiety score	4 (1–8)	6 (3–10)	4736	0.005
Depression score	4 (2–8)	5 (3–9)	4871	0.01
ORA score	4 (2–5)	3 (2–5)	6359	0.763
NORA score	3 (1–5)	4 (1–5)	5574	0.204
IR score	11 (8–13.5)	11 (7–13)	6571	0.480
Total DUREL score	18 (12–22.5)	18 (12–22)	6267	0.906

ORA score = Organized religious activity score; NORA = Non-organized religious activity score; IR = Intrinsic religiosity score; DUREL = The Duke University Religion Index.

**Table 8:** Distribution of respondents across depression (PHQ-9) and anxiety (GAD-7) symptom categories by religiosity level (DUREL), with Chi-squared test results (*n* = 260).

Variable	Category	Low Religiosity * (*n* = 73)	Moderate and High Religiosity ** (*n* = 187)	*χ* ^2^	*p*
Depression symptoms	Minimal and mild	64	140	4.36	0.037
	Moderate, moderately severe, and severe	9	47		
Anxiety symptoms	Minimal and mild	58	137	0.77	0.381
	Moderate and severe	15	50		

* Respondents with a total DUREL score of 12 or lower; ** Respondents with a total DUREL score of 13 or higher. Chi-square (χ^2^) tests were conducted using Yates’ continuity correction for 2 × 2 contingency tables.

**Table 9:** Spearman’s rank correlation coefficients between depression (PHQ-9), anxiety (GAD-7), and DUREL subscale scores (n = 260).

Variables	(1)	(2)	(3)	(4)	(5)	(6)
(1) Depression score						
(2) Anxiety score	0.75 *					
(3) Organized religious activity (ORA) score	0.05	0.08				
(4) Non-organized religious activity (NORA) score	0.07	0.07	0.63 *			
(5) Intrinsic religiosity (IR) score	0.07	0.05	0.62 *	0.62 *		
(6) Total DUREL score	0.07	0.07	0.81 *	0.81 *	0.93 *	

DUREL = The Duke University Religion Index. * Correlation is significant at the 0.001 level (two-tailed).

## Data Availability

The original contributions presented in this study are included in the article/Supplementary Material. Further inquiries can be directed to the corresponding authors.

## Supplementary Materials

The following supporting information can be downloaded at https://www.mdpi.com/article/10.3390/diagnostics16010172/s1, File S1: Participant Information Sheet; File S2: Consent Form and Questionnaire; File S3: Raw Data.

## Abbreviations

The following abbreviations are used in this manuscript:

GAD-7	Generalized Anxiety Disorder-7
PHQ-9	Patient Health Questionnaire 9
DUREL	Duke University Religion Index
ORA	Organized religious activity
NORA	Non-organized religious activity
IR	Intrinsic religiosity
BMI	Body mass Index
GPA	Grade Point Average

## References

[1] Santomauro D.F., Mantilla Herrera A.M., Shadid J., Zheng P., Ashbaugh C., Pigott D.M., Abbafati C., Adolph C., Amlag J.O., Aravkin A.Y. (2021). Global Prevalence and Burden of Depressive and Anxiety Disorders in 204 Countries and Territories in 2020 Due to the COVID-19 Pandemic. Lancet.

[2] World Health Organisation (WHO) (2025). Mental Disorders. https://www.who.int/news-room/fact-sheets/detail/mental-disorders.

[3] Hohls J.K., König H.-H., Quirke E., Hajek A. (2021). Anxiety, Depression and Quality of Life—A Systematic Review of Evidence from Longitudinal Observational Studies. Int. J. Environ. Res. Public Health.

[4] Sundqvist A.J.E., Hemberg J., Nyman-Kurkiala P., Ness O. (2024). Are Educational Transitions Related to Young People’s Loneliness and Mental Health: A Systematic Review. Int. J. Adolesc. Youth.

[5] Yuan F., Peng S., Khairani A.Z., Liang J. (2024). A Systematic Review and Meta-Analysis of the Efficacy of Physical Activity Interventions among University Students. Sustainability.

[6] Oftedal S., Fenton S., Hansen V., Whatnall M.C., Ashton L.M., Haslam R.L., Hutchesson M.J., Duncan M.J. (2024). Changes in Physical Activity, Diet, Sleep, and Mental Well-Being When Starting University: A Qualitative Exploration of Australian Student Experiences. J. Am. Coll. Health.

[7] Carrard V., Berney S., Bourquin C., Ranjbar S., Castelao E., Schlegel K., Gaume J., Bart P.-A., Schmid Mast M., Preisig M. (2024). Mental Health and Burnout during Medical School: Longitudinal Evolution and Covariates. PLoS ONE.

[8] Nair M., Moss N., Bashir A., Garate D., Thomas D., Fu S., Phu D., Pham C. (2023). Mental Health Trends among Medical Students. Bayl. Univ. Med. Cent. Proc..

[9] Peng P., Hao Y., Liu Y., Chen S., Wang Y., Yang Q., Wang X., Li M., Wang Y., He L. (2023). The Prevalence and Risk Factors of Mental Problems in Medical Students during COVID-19 Pandemic: A Systematic Review and Meta-Analysis. J. Affect. Disord..

[10] Ayubi E., Bashirian S., Jenabi E., Barati M., Khazaei S. (2023). Stress, Anxiety and Depression among Medical Students during COVID-19 Pandemic: A Systematic Review and Meta-Analysis. Pers. Med. Psychiatry.

[11] Mengesha A.K., Misker M.F., Tadesse S.A. (2025). Identifying Factors Contributing to Depression and Anxiety among Medical Students: A Multicenter Cross-Sectional Study. Sci. Rep..

[12] Daniali H., Martinussen M., Flaten M.A. (2023). A Global Meta-Analysis of Depression, Anxiety, and Stress before and during COVID-19. Health Psychol..

[13] Paiva U., Cortese S., Flor M., Moncada-Parra A., Lecumberri A., Eudave L., Magallón S., García-González S., Sobrino-Morras Á., Piqué I. (2025). Prevalence of Mental Disorder Symptoms among University Students: An Umbrella Review. Neurosci. Biobehav. Rev..

[14] Moreira De Sousa J., Moreira C.A., Telles-Correia D. (2018). Anxiety, Depression and Academic Performance: A Study Amongst Portuguese Medical Students Versus Non-Medical Students. Acta Médica Port..

[15] AlSamhori J.F., Kakish D.R.K., AlSamhori A.R.F., AlSamhori A.F., Hantash N.R.A., Swelem A.F.A., Abu-Suaileek M.H.A., Arabiat H.M., Altwaiqat M.A., Banimustafa R. (2024). Anxiety, Depression, and Insomnia among Medical and Non-Medical Students in Jordan: A Cross-Sectional Study. Middle East Curr. Psychiatry.

[16] Mirza A.A., Milaat W.A., Ramadan I.K., Baig M., Elmorsy S.A., Beyari G.M., Halawani M.A., Azab R.A., Zahrani M.T., Khayat N.K. (2021). Depression, Anxiety and Stress among Medical and Non-Medical Students in Saudi Arabia: An Epidemiological Comparative Cross-Sectional Study. Neurosciences.

[17] Bacchi S., Licinio J. (2015). Qualitative Literature Review of the Prevalence of Depression in Medical Students Compared to Students in Non-Medical Degrees. Acad. Psychiatry.

[18] Amarasuriya S.D., Jorm A.F., Reavley N.J. (2015). Perceptions and Intentions Relating to Seeking Help for Depression among Medical Undergraduates in Sri Lanka: A Cross-Sectional Comparison with Non-Medical Undergraduates. BMC Med. Educ..

[19] Wielewska M.K., Godzwon J.M., Gargul K., Nawrocka E., Konopka K., Sobczak K., Rudnik A., Zdun-Ryzewska A. (2022). Comparing Students of Medical and Social Sciences in Terms of Self-Assessment of Perceived Stress, Quality of Life, and Personal Characteristics. Front. Psychol..

[20] Vidović S., Kotromanović S., Pogorelić Z. (2024). Depression, Anxiety, and Stress Symptoms Among Students in Croatia During the COVID-19 Pandemic: A Systematic Review. J. Clin. Med..

[21] Milić J., Škrlec I., Milić Vranješ I., Podgornjak M., Heffer M. (2019). High Levels of Depression and Anxiety among Croatian Medical and Nursing Students and the Correlation between Subjective Happiness and Personality Traits. Int. Rev. Psychiatry.

[22] Vidović S., Rakić N., Kraštek S., Pešikan A., Degmečić D., Zibar L., Labak I., Heffer M., Pogorelić Z. (2025). Sleep Quality and Mental Health Among Medical Students: A Cross-Sectional Study. J. Clin. Med..

[23] Vidović S., Degmečić D., Drenjančević I., Labak I., Pešikan A., Kolak E., Kraštek S., Heffer M. (2025). Mental Health and Physical Activity Among Medical Students: A Cross-Sectional Study. Psychiatry Int..

[24] Rosmarin D.H., Kaufman C.C., Ford S.F., Keshava P., Drury M., Minns S., Marmarosh C., Chowdhury A., Sacchet M.D. (2022). The Neuroscience of Spirituality, Religion, and Mental Health: A Systematic Review and Synthesis. J. Psychiatr. Res..

[25] Koenig H.G., Carey L.B. (2024). Religion, Spirituality and Health Research: Warning of Contaminated Scales. J. Relig. Health.

[26] Aggarwal S., Wright J., Morgan A., Patton G., Reavley N. (2023). Religiosity and Spirituality in the Prevention and Management of Depression and Anxiety in Young People: A Systematic Review and Meta-Analysis. BMC Psychiatry.

[27] Sallquist J., Eisenberg N., French D.C., Purwono U., Suryanti T.A. (2010). Indonesian Adolescents’ Spiritual and Religious Experiences and Their Longitudinal Relations with Socioemotional Functioning. Dev. Psychol..

[28] Rasic D., Asbridge M., Kisely S., Langille D. (2013). Longitudinal Associations of Importance of Religion and Frequency of Service Attendance with Depression Risk among Adolescents in Nova Scotia. Can. J. Psychiatry.

[29] Paunesku D., Ellis J., Fogel J., Kuwabara S.A., Gollan J., Gladstone T., Reinecke M., Van Voorhees B.W. (2008). Clusters of Behaviors and Beliefs Predicting Adolescent Depression: Implications for Prevention. J. Cogn. Behav. Psychother. Off. J. Int. Inst. Adv. Stud. Psychother. Appl. Ment. Health.

[30] Smokowski P.R., Guo S., Evans C.B.R., Wu Q., Rose R.A., Bacallao M., Cotter K.L. (2017). Risk and Protective Factors across Multiple Microsystems Associated with Internalizing Symptoms and Aggressive Behavior in Rural Adolescents: Modeling Longitudinal Trajectories from the Rural Adaptation Project. Am. J. Orthopsychiatry.

[31] Smokowski P.R., Guo S., Rose R., Evans C.B.R., Cotter K.L., Bacallao M. (2014). Multilevel Risk Factors and Developmental Assets for Internalizing Symptoms and Self-Esteem in Disadvantaged Adolescents: Modeling Longitudinal Trajectories from the Rural Adaptation Project. Dev. Psychopathol..

[32] Lucchetti G., Koenig H.G., Lucchetti A.L.G. (2021). Spirituality, Religiousness, and Mental Health: A Review of the Current Scientific Evidence. World J. Clin. Cases.

[33] Birhan B., Eristu N. (2023). Positive Religious Coping and Associated Factors Among Participants with Severe Mental Illness Attending Felege Hiwot Comprehensive Specialized Hospital, Bahir Dar, Ethiopia, 2021. Psychol. Res. Behav. Manag..

[34] Malinakova K., Vyvleckova L., Novak L. (2024). Religiosity/Spirituality and Mental Health: The Moderating Role of Sensory Processing Sensitivity. Humanit. Soc. Sci. Commun..

[35] Mancini C.J., Quilliam V., Camilleri C., Sammut S. (2023). Spirituality and Negative Religious Coping, but Not Positive Religious Coping, Differentially Mediate the Relationship between Scrupulosity and Mental Health: A Cross-Sectional Study. J. Affect. Disord. Rep..

[36] Spitzer R.L., Kroenke K., Williams J.B.W., Löwe B. (2006). A Brief Measure for Assessing Generalized Anxiety Disorder: The GAD-7. Arch. Intern. Med..

[37] Löwe B., Decker O., Müller S., Brähler E., Schellberg D., Herzog W., Herzberg P.Y. (2008). Validation and Standardization of the Generalized Anxiety Disorder Screener (GAD-7) in the General Population. Med. Care.

[38] Milić J., Skitarelić N., Majstorović D., Zoranić S., Čivljak M., Ivanišević K., Marendić M., Mesarić J., Puharić Z., Neuberg M. (2024). Levels of Depression, Anxiety and Subjective Happiness among Health Sciences Students in Croatia: A Multi-Centric Cross-Sectional Study. BMC Psychiatry.

[39] Kroenke K., Spitzer R.L., Williams J.B.W., Löwe B. (2010). The Patient Health Questionnaire Somatic, Anxiety, and Depressive Symptom Scales: A Systematic Review. Gen. Hosp. Psychiatry.

[40] Koenig H.G. (2012). Religion, Spirituality, and Health: The Research and Clinical Implications. ISRN Psychiatry.

[41] Koenig H.G., Büssing A. (2010). The Duke University Religion Index (DUREL): A Five-Item Measure for Use in Epidemological Studies. Religions.

[42] Murgić L., Mihaljević S., Sović S., Aukst-Margetić B., Pavleković G. (2018). Validation of the Croatian Version of the Duke Religion Index (DUREL-Hr) among Medical School Students. Coll. Antropol..

[43] Lin Y.-K., Saragih I.D., Lin C.-J., Liu H.-L., Chen C.-W., Yeh Y.-S. (2024). Global Prevalence of Anxiety and Depression among Medical Students during the COVID-19 Pandemic: A Systematic Review and Meta-Analysis. BMC Psychol..

[44] Dodin Y., Obeidat N., Dodein R., Seetan K., Alajjawe S., Awwad M., Adwan M., Alhawari A., ALkatari A., Alqadasi A.A. (2024). Mental Health and Lifestyle-Related Behaviors in Medical Students in a Jordanian University, and Variations by Clerkship Status. BMC Med. Educ..

[45] Huber A., Rabl L., Höge-Raisig T., Höfer S. (2023). Well-Being, Mental Health, and Study Characteristics of Medical Students before and during the Pandemic. Behav. Sci..

[46] Eley D.S., Slavin S.J. (2024). Medical Student Mental Health—The Intransigent Global Dilemma: Contributors and Potential Solutions. Med. Teach..

[47] Ahmed I., Hazell C.M., Edwards B., Glazebrook C., Davies E.B. (2023). A Systematic Review and Meta-Analysis of Studies Exploring Prevalence of Non-Specific Anxiety in Undergraduate University Students. BMC Psychiatry.

[48] Hantsoo L., Jagodnik K.M., Novick A.M., Baweja R., Di Scalea T.L., Ozerdem A., McGlade E.C., Simeonova D.I., Dekel S., Kornfield S.L. (2023). The Role of the Hypothalamic-Pituitary-Adrenal Axis in Depression across the Female Reproductive Lifecycle: Current Knowledge and Future Directions. Front. Endocrinol..

[49] Gao W., Ping S., Liu X. (2020). Gender Differences in Depression, Anxiety, and Stress among College Students: A Longitudinal Study from China. J. Affect. Disord..

[50] Graves B.S., Hall M.E., Dias-Karch C., Haischer M.H., Apter C. (2021). Gender Differences in Perceived Stress and Coping among College Students. PLoS ONE.

[51] Johnson D.P., Whisman M.A. (2013). Gender Differences in Rumination: A Meta-Analysis. Personal. Individ. Differ..

[52] Stimac Grbic D., Pavic Simetin I., Muslic L., Music Milanovic S. (2023). Suicide Prevention-a Public Health Priority in the Republic of Croatia. Eur. J. Public Health.

[53] Kroenke K., Spitzer R.L., Williams J.B.W. (2001). The PHQ-9: Validity of a Brief Depression Severity Measure. J. Gen. Intern. Med..

[54] González-Martín A.M., Aibar-Almazán A., Rivas-Campo Y., Castellote-Caballero Y., Carcelén-Fraile M.D.C. (2023). Mindfulness to Improve the Mental Health of University Students. A Systematic Review and Meta-Analysis. Front. Public Health.

[55] Cerolini S., Zagaria A., Franchini C., Maniaci V.G., Fortunato A., Petrocchi C., Speranza A.M., Lombardo C. (2023). Psychological Counseling among University Students Worldwide: A Systematic Review. Eur. J. Investig. Health Psychol. Educ..

[56] Chye S.M., Kok Y.Y., Chen Y.S., Er H.M. (2024). Building Resilience among Undergraduate Health Professions Students: Identifying Influencing Factors. BMC Med. Educ..

[57] Bonelli R., Dew R.E., Koenig H.G., Rosmarin D.H., Vasegh S. (2012). Religious and Spiritual Factors in Depression: Review and Integration of the Research. Depress. Res. Treat..

[58] Forouhari S., Hosseini Teshnizi S., Ehrampoush M.H., Mazloomy Mahmoodabad S.S., Fallahzadeh H., Tabei S.Z., Nami M., Mirzaei M., Namavar Jahromi B., Hosseini Teshnizi S.M. (2019). Relationship between Religious Orientation, Anxiety, and Depression among College Students: A Systematic Review and Meta-Analysis. Iran. J. Public Health.

[59] Hazdovac Bajić N. (2022). Being Nonreligious in Croatia: Managing Belonging and Non-Belonging. Religions.

[60] Bockrath M.F., Pargament K.I., Wong S., Harriott V.A., Pomerleau J.M., Homolka S.J., Chaudhary Z.B., Exline J.J. (2022). Religious and Spiritual Struggles and Their Links to Psychological Adjustment: A Meta-Analysis of Longitudinal Studies. Psychol. Relig. Spiritual..

[61] Gwin S., Branscum P., Taylor L., Cheney M., Maness S.B., Frey M., Zhang Y. (2020). Associations Between Depressive Symptoms and Religiosity in Young Adults. J. Relig. Health.

[62] Dolcos F., Hohl K., Hu Y., Dolcos S. (2021). Religiosity and Resilience: Cognitive Reappraisal and Coping Self-Efficacy Mediate the Link between Religious Coping and Well-Being. J. Relig. Health.

[63] Osborn T.G., Li S., Saunders R., Fonagy P. (2022). University Students’ Use of Mental Health Services: A Systematic Review and Meta-Analysis. Int. J. Ment. Health Syst..

[64] Graça L., Brandão T. (2024). Religious/Spiritual Coping, Emotion Regulation, Psychological Well-Being, and Life Satisfaction among University Students. J. Psychol. Theol..

