# Supplementary material for: Assessment of Anxiety and Depression Symptoms Among Medical Students and Their Association with Religiosity: A Cross-Sectional Study
Source: Diagnostics (Basel). 2026 Jan 5;16(1):172. doi: 10.3390/diagnostics16010172 (PMC12785332; doi:10.3390/diagnostics16010172)
Supplement: Supplementary file 1 [file diagnostics-16-00172-s001.zip › Supplement File S1. Paricipant information sheet .pdf]

Participant Information Sheet

Dear prospective participant,

You are invited to take part in a scientific study entitled *"Screening and Assessment of Anxiety and Depression Symptoms among Medical Students and Their Association with Religiosity: A Cross-Sectional Study".*

Your participation should be based on a clear understanding of the study's aims, methods, and procedures, as well as the potential benefits and risks to you as a participant. Therefore, we kindly ask you to read this information carefully before deciding whether to take part.

DESCRIPTION OF THE STUDY AND OBJECTIVES

To our knowledge, no study has examined the prevalence of depression, anxiety, and burnout symptoms among medical students in Croatia, nor their association with religiosity.

The aims of the study are:

- To assess the prevalence of anxiety symptoms using the Generalized Anxiety Disorder 7-item scale (GAD-7).
- To assess the prevalence of depression symptoms using the Patient Health Questionnaire-9 (PHQ-9).
- To measure religiosity using the Duke University Religion Index (DUREL).
- To explore the association between symptoms of depression and anxiety and students' spirituality/religiosity.

YOUR ROLE AS A PARTICIPANT

Your primary task will be to complete a questionnaire, which is **entirely anonymous**. Researchers will follow all procedures for protecting personal data in medical research. At no point will you be asked to provide your name, surname, or email address.

The questionnaire consists of five sections (40 questions) and will take approximately 10 minutes to complete. The sections include:

1. Sociodemographic characteristics.
2. Prevalence of anxiety symptoms – *The Generalized Anxiety Disorder 7-item Scale (GAD-7)*
3. Prevalence of depression symptoms – *The Patient Health Questionnaire-9 (PHQ-9)*
4. Religiosity – *The Duke University Religion Index (DUREL)*

You may withdraw from the study at any time, without providing any reason and without any negative consequences. Your decision to withdraw will not affect your rights, your academic status, or your relationship with the researchers or the institution. Any data collected from you up to the point of withdrawal will remain confidential and will only be used in an anonymized form, unless you specifically request otherwise.

The questionnaire will be administered via Google Forms, and the link is provided at the end of this document.

ETHICAL APPROVAL

This study has been reviewed and approved by the Ethics Committee of the Faculty of Medicine, Osijek, after thorough evaluation of the research proposal and accompanying documentation.

The study will be conducted in accordance with all applicable guidelines to ensure proper research procedures and participant safety, including the *"Good Clinical Practice Guidelines"* and the *"Declaration of Helsinki."*

CONSENT TO PARTICIPATE

Your decision to participate is entirely voluntary.

You will be considered to have given consent if, after reading this Information Sheet, you follow the questionnaire hyperlink (Google Forms) and confirm your participation by selecting the "Yes" option in the consent statement at the beginning of the questionnaire:

"I have read the Information for the Participant for the scientific research entitled 'Screening of Anxiety and Depression Symptoms among Medical Students and Their Association with Religiosity: A Cross-Sectional Study'. I understand that the research is anonymous, my participation is voluntary, and I can withdraw from participation at any time without giving reasons and without any consequences. I want and agree to participate in the mentioned scientific research."

QUESTIONNAIRE LINK

https://forms.gle/atPmZCLGBerHn7hS8

Thank you for reading this document and considering your participation in this scientific study.
