# Supplementary material for: Assessment of Anxiety and Depression Symptoms Among Medical Students and Their Association with Religiosity: A Cross-Sectional Study
Source: Diagnostics (Basel). 2026 Jan 5;16(1):172. doi: 10.3390/diagnostics16010172 (PMC12785332; doi:10.3390/diagnostics16010172)
Supplement: Supplementary file 1 [file diagnostics-16-00172-s001.zip › Supplement File S2. Questionnaire_REVISED.pdf]

Questionnaire

I have read the Information for the Participant for the scientific research entitled " *Screening and Assessment of Anxiety and Depression Symptoms among Medical Students and Their Association with Religiosity: A Cross-Sectional Study*". I understand that the research is anonymous, my participation is voluntary, and I can withdraw from participation at any time without giving reasons and without any consequences. I want and agree to participate in the mentioned scientific research.

a) Yes
b) No

1. Gender
a) Male
b) Female
c) Other

2. Your age __________________ (years)

3. Your weight ________________ (kg)

4. Your height __________________ (cm)

5. Year of study _____________________.

6. GPA of the last academic year (If you are in the first year of college, enter the average of the final year of high school; two decimals) ________________.

7. Place of residence
a) Urban
b) Rural

8. Type of accommodation
a) Student dormitory
b) Rented apartment/room
c) Own accommodation
d) Living with parents

9. I live
a) Alone
b) With a roommate
c) With family

10. Relationship status
a) Single
b) In a relationship

11. I consider my current financial/material status
a) Far below average
b) Below average
c) Average
d) Above average
e) Far above average

12. My parents
a) Live together
b) Do not live together

13. Mother's education
a) Primary school
b) Secondary education
c) Higher education or vocational education

14. Father's education
a) Primary school
b) Secondary education
c) Higher education or vocational education

15. Smoking
a) Current smoker
b) Former smoker
c) Non-smoker

16. How often do you consume alcohol? * (Mark one option)
a) Never
b) Sometimes
c) Once a week
d) Twice a week
e) 3 or more times a week

17. Do you consume psychoactive substances?
a) Yes
b) No

18. Considering your current mental health, do you think you would benefit from professional advice and help from a psychologist/psychiatrist?
a) Yes
b) Sometimes
c) No

19. Have you sought help from a psychologist or psychiatrist?
a) Yes
b) No

Generalized Anxiety Disorder-7 (GAD-7) instrument

20. Over the **last 2 weeks**, how often have you been bothered by the following problems?

	Not at all	Several days	More than half the days	Nearly every day
1. Feeling nervous, anxious or on edge				
2. Not being able to stop or control worrying				
3. Worrying too much about different things				
4. Trouble relaxing				
5. Being so restless that it is hard to sit still				
6. Becoming easily annoyed or irritable				
7. Feeling afraid as if something awful might happen				

Patient Health Questionnaire 9 (PHQ-9)

21. Over the last 2 weeks, how often have you been bothered by any of the following problems?

	Not at all	Several days	More than half the days	Nearly every day
1. Little interest or pleasure in doing things				
2. Feeling down, depressed, or hopeless				
3. Trouble falling or staying asleep, or sleeping too much				
4. Feeling tired or having little energy				
5. Poor appetite or overeating				
6. Feeling bad about yourself — or that you are a failure or have let yourself or your family down				
7. Trouble concentrating on things, such as reading the newspaper or watching television				
8. Moving or speaking so slowly that other people could have noticed? Or the opposite — being so fidgety or restless that you have been moving around a lot more than usual				
9. Thoughts that you would be better off dead or of hurting yourself in some way				

The Duke University Religion Index (DUREL)

22. How often do you attend church or other religious meetings?
1) Never
2) Once a year or less
3) Several times a year
4) Several times a month

5) Once a week
6) More than once a week

23. How often do you spend time in private religious activities, such as prayer, meditation or Bible study?
1) Rarely or never
2) Several times a month
3) Once a week
4) Twice or more times a week
5) Every day
6) More than once a day

The following section contains 3 statements about religious belief or experience. Please mark the extent to which each statement is true or not true for you.

24. In my life, I experience the presence of the Divine (*i.e.*, God)
1) Definitely *not* true
2) Tends *not* to be true
3) Unsure
4) Tends to be true
5) Definitely true of me

25. My religious beliefs are what really lie behind my whole approach to life
1) Definitely *not* true
2) Tends *not* to be true
3) Unsure
4) Tends to be true
5) Definitely true of me

26. I try hard to carry my religion over into all other dealings in life
1) Definitely *not* true
2) Tends *not* to be true
3) Unsure
4) Tends to be true
5) Definitely true of me
